# Therapeutic Application of Betalains: A Review

**DOI:** 10.3390/plants9091219

**Published:** 2020-09-17

**Authors:** Elaheh Madadi, Sahand Mazloum-Ravasan, Jae Sik Yu, Ji Won Ha, Hamed Hamishehkar, Ki Hyun Kim

**Affiliations:** 1 Biotechnology Research Center and Student’s Research Committee, Tabriz University of Medical Sciences, Tabriz 51368, Iran; madadie@tbzmed.ac.ir; 2 Research Center for Pharmaceutical Nanotechnology, Tabriz University of Medical Sciences, Tabriz 51368, Iran; Perzhad.mazloumi@gmail.com; 3 School of Pharmacy, Sungkyunkwan University, Suwon 16419, Korea; jsyu@bu.edu (J.S.Y.); ellenha2@gmail.com (J.W.H.); 4 Drug Applied Research Center, Tabriz University of Medical Sciences, Tabriz 51368, Iran

**Keywords:** betalain, cancer, natural product, beetroot, *Beta vulgaris*

## Abstract

Anthocyanins, betalains, riboflavin, carotenoids, chlorophylls and caramel are the basic natural food colorants used in modern food manufacture. Betalains, which are composed of red–violet betacyanin and yellow betaxanthins, are water-soluble pigments that color flowers and fruits. Betalains are pigments primarily produced by plants of the order Caryophyllales. Because of their anti-inflammatory, cognitive impairment, anticancer and anti-hepatitis properties, betalains are useful as pharmaceutical agents and dietary supplements. Betalains also exhibit antimicrobial and antimalarial effects, and as an example, betalain-rich *Amaranthus spinosus* displays prominent antimalarial activity. Studies also confirmed the antidiabetic effect of betalains, which reduced glycemia by 40% without causing weight loss or liver impairment. These findings show that betalain colorants may be a promising alternative to the synthetic dyes currently used as food additives.

## 1. Introduction

Vegetables and phytochemicals markedly decrease the risk of different degenerative and chronic diseases [1] such as colorectal cancer, which is a significant cause of death worldwide; however, only few individuals use a traditional diet including fruits and vegetables. Recently, the use of natural food colorants has increased mainly owing to their low toxicity, environmental safety and renewable vegetable origin [2]. In addition to enhancing the appearance of food, natural colorants have bioactive properties that protect the plant in which they are contained against environmental stimuli caused by infections with fungi, insects or microorganisms. Furthermore, some colorants may be beneficial to human health [3]. Food colorants such as betalains have chemoprotective effects that combat oxidative stress and balance oxidants and antioxidants in the body. The Chenopodiaceae family includes two classes of vegetables, containing Swiss chard (*Beta vulgaris* L. var. *cicla*; BVc) and beetroot (*B. vulgaris* var. *rubra* L.; BVr), which have been a part of the traditional western diet [4].

The powder or extract form of betanin, a natural pigment, is an antioxidant used in the food industry. The antioxidant activity of betanin in biologic lipid environments has been indicated in human macromolecules such as membranes, low-density lipoproteins (LDL) and whole cells [5]. Moreover, betanin exerts anti-inflammatory effect and protects hepatic functions in human cells. The compound regulates redox signaling pathways mediated by the inflammatory response in cultured endothelial cells and exerts antiproliferative effects on human tumor cell lines [6]. Specifically, in both healthy liver and tumoral human hepatic cell lines, betanin induces the translocation of the an antioxidant response element called erythroid 2-related factor 2 (*Nrf2*) from the cytosol to the nuclear compartment, known to conduct mRNA and protein levels of detoxifying/antioxidant enzymes including *GSTM, GSTP, GSTA (glutathione S-transferases), GSTT,* HO– (hemeoxygenase-1) and *NQO1 (NAD(P)H quinone dehydrogenase 1*), thereby, exerting hepatoprotective and anticarcinogenic effects [7,8]. Various studies related to isolation of betanin, involving comprehensive steps and procedures to extract the purified compound from plant sources including complex food matrices such as beets. Among the purification studies of betanin, it is said that chromatographic methods, including high-efficiency liquid chromatography, applying reverse phase columns provide most efficient results [9]. However, no studies have been found evaluating the stability of this molecule during storage conditions or its antioxidant ability after purification and during storage [10].

In the food industry, synthetic antioxidants are added to fatty foods, especially meats, for delaying oxidative processes that cause sensorial changes, decreases in nutritional values and formation of secondary compounds that are potentially harmful to health during storage 11]. However, the synthetic antioxidants butylated hydroxytoluene (BHT) and butylated hydroxyanisole (BHA) may cause harmful effects to human health, as they have been reported to be potential tumor promoters succeeding long-term administration to animals [11]. Therefore, these synthetic antioxidants have been replaced with natural antioxidants extracted from food [11,12].

**Figure 1:**
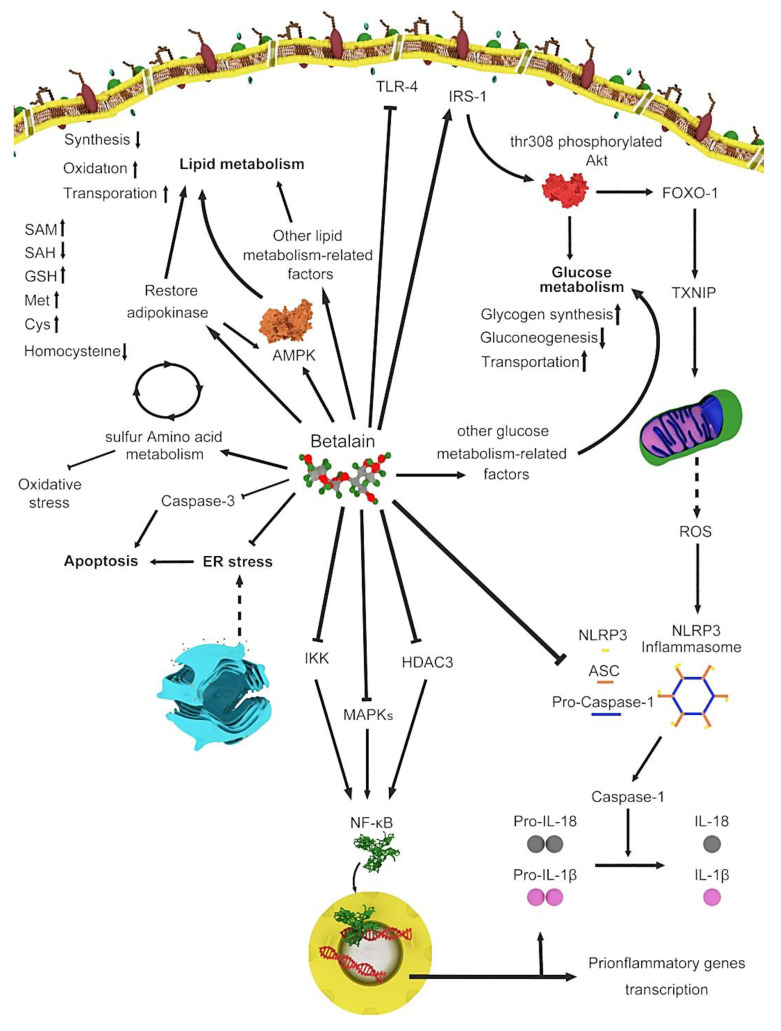
Various genes and routes involved in betalain metabolism.

Betalain-rich extracts from food sources has been investigated for the antitumoral potential in animal models and cancer cell lines [4,5,6]. Betanin, the original nutritional betacyanin, show significant inhibition to the growth of tumor cells of the stomach, breast, lung, colon and central nervous system [7]; induce apoptosis in K562 human myeloid leukemia cells; and weakly exhibit epigenome-regulated gene expression in MCF-7 breast cancer cells. However, the potential antiproliferative, chemopreventive and epigenetic activities of betaxanthins are yet to be investigated [8]. Recently, the focus has shifted to the usage of natural products to improve human health instead of prevention diseases [9]. Thus, the number of studies on the application of betalains in medical sciences is increasing. Therefore, a narrative review of the therapeutic uses of betalains and the genes involved in betalain metabolism (Figure 1) may help future investigations regarding the advantages of natural products. Because of the importance of issue, few interesting reviews articles have been published very recently [11,12,13]. In this review, we tried to offer updated data in different therapeutic classification with focus to the molecular mechanisms of betalains.

## 2. Taxonomy

Betalains contain two classes of pigments, namely yellow betaxanthins and red betacyanins [14]. Red beetroot (BVr) extract is a group of betalains with significant antioxidant activity, which is attributed to betalamic acid, as determined by the 2,2′-azino-bis (3-ethylbenzothiazoline-6-sulfonic acid (ABTS) and ferric reducing antioxidant power methods [15]. Beet is taxonomically classified as the genus *Beta* of the subfamily Chenopodiaceae, subclass Caryophyllidae and class Dicotyledonae [16]. Based on its morphologic characteristics, the genus *Beta* includes two groups, namely cultivated and wild maritime beets. In the wild maritime group, a unique species called sea beets (*B. vulgaris maritima*) is the ancestral form of all the remaining species. The cultivated group consists of sugar beets (*B. vulgaris saccharifera*), leaf beets (*B. vulgaris cicla*), forage beets (*B. vulgaris crassa*) and garden beets (*B. vulgaris rubra*) [16]. A list of betalain-producing plants is provided in Table 1.

## 3. Therapeutic Effect of Betalains

### 3.1. Antidiabetic Activity

Yanardag et al. [27] identified a low level of blood sugar in diabetic rats administered BVc extract. Further investigation found that glycemia in these rats was reduced to 40% without causing weight loss or hepatic impairment [28].

**Figure 2:**
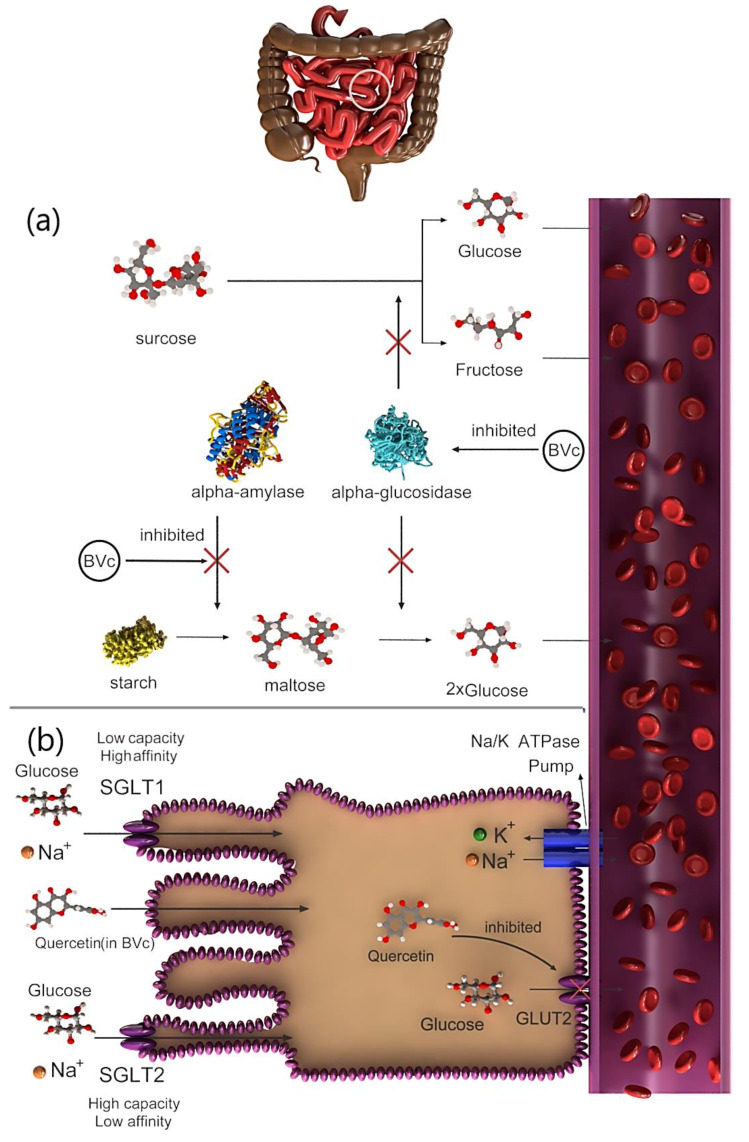
Mechanism of hypoglycemic function of *Beta vulgaris* var. *cicla* L. (BVc) by (**a**) *alpha-amylase* and *alpha-glucosidase* to control glucose in diabetic patients and (**b**) inhibiting glucose transporter isoform 2 (GLUT2).

The hypoglycemic action of the extract is attributed to saponins, which prevent glycogenolysis and gluconeogenesis [29]. Therefore, the molecular pathways that affect this hypoglycemic mechanism must be thoroughly examined. Several studies showed that the hypoglycemic activity of the BVc extract, which is mediated via prevention of glucose transporters, may be caused by flavonoids. For example, quercetin, which is found in BVc, exhibited antidiabetic effects by preventing the action of the intestinal glucose transporter GLUT2 (Figure 2b) [30]. Another hypoglycemic mechanism is the inhibition of α-amylase and α-glucosidase activities by flavonoids [31]. For example, two flavanol glycosides isolated from *Salsola kali* actively inhibited *α-amylase* activity [32]. The digestion and absorption of carbohydrates can be delayed by inhibiting *α-amylase* activity, consequently suppressing postprandial hyperglycemia (Figure 2a) [33]. Vitexin-2-*O*-glycoside, a C-glycosyl flavone-containing vitexin found in the leaves and seeds of BVc, strongly inhibits *α-glucosidase* (Figure 2a) [34]. This finding suggests that α-glucosidase inhibition serves as the primary mechanism underlying the hypoglycemic effect previously observed in diabetic rats [28,34]. In 2014, more than 1.9 billion adults worldwide were estimated to be overweight and more than 600 million of whom were obese. Intake of high-energy foods with low fat is the leading inducer of obesity and overweightness [35]. Type 2 diabetes is a form of chronic diabetes triggered by hyperglycemia, and it leads to impaired insulin secretion, insulin action or both. In contrast, obesity is characterized by chronic low-grade inflammation in the adipose tissue, liver and skeleton, leading to areas of hypoxia in adipose tissue [36]. Nutritional therapy and glucose monitoring, including diet control, are suggested as interventions to monitoring the blood sugar in type 2 diabetes. The antihypertensive activities of quinoa and amaranth have been evaluated using laboratory enzymatic methods, and their anti-obesity effects have been studied in obese and hyperglycemic mouse models [37]. Glucosidase and pancreatic lipase are essential enzymes for breaking down complex carbohydrates and absorption of triglyceride lipids. The use of bioactive agents in foods to control both enzymes may have potential benefits in regulating blood sugar and weight and consequently manage obesity and type 2 diabetes. The phenolic content of quinoa inhibits α-glucosidase and pancreatic lipase activities [38]. The most common phytoecdysteroid, 20HE, is extracted from quinoa seeds, and it significantly reduces fasting blood sugar in obese mice. In addition, mice fed 20HE-enriched quinoa decreased mRNA levels of various genes related to inflammation (*monocyte chemotactic protein 1*, *CD68*) and reduced insulin resistance [37]. Quinoa diet is known to show reversed effects of HF-induced depletion of unbroken proteins in mice. In the study, male Wistar rats fed with amaranth seeds was shown to have significantly lower plasma MDA levels and higher antioxidant enzyme activity than the control rats. Amaranth seeds can act as a medium to protect against obesity caused by fructose and diabetes [39]. Amaranth seed and its oil showed reduced serum glucose level and increased serum insulin levels in rats with diabetes. Therefore, amaranth seed is useful for correcting blood sugar level and preventing diabetic side effects. However, the components responsible for its anti-obesity and antidiabetic activities are still unknown. The anti-obesity and antidiabetic activities of quinoa and amaranth have been investigated mostly in in vitro and in vivo experiments, whereas clinical studies have been limited. Therefore, the effect of diets containing quinoa and amaranth must be investigated in humans [37].

### 3.2. Cardiovascular Disease (CVD)

**Figure 3:**
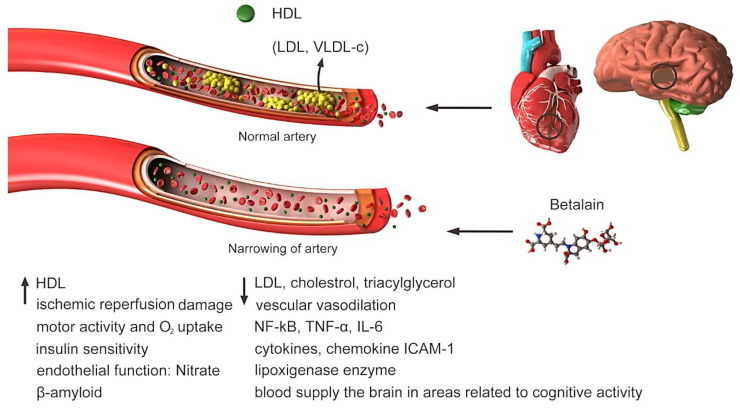
Highly concentrated low-density lipoprotein (LDL) is associated with increased cholesterol deposition in the walls of blood vessels and atherosclerosis (susceptibility to cardiovascular disease) and should be tried to reduce its level in the blood. Clogged or narrowed arteries can block blood flow to the heart, brain or other organs. This can lead to stroke, heart attack or even heart failure. Betalain reduces blood LDL, increases HDL and vascular vasodilation. Other factors that increase or decrease under the influence of betalain are shown in Figure.

CVD is the leading cause of death and disability worldwide. Unhealthy diet is considered as one of the most significant risk factors for CVD [40]. Total cholesterol, LDL cholesterol (LDL-c), and triglyceride concentrations are risk markers of CVD (Figure 3). The effects of dietary quinoa (quinoa contains a marked concentration of betalain: 630.4 mg/100 g dry portion) on risk parameters of CVD were evaluated after 30 days of consumption in 22 students between 18 and 45 years old. Approximately 42.2% and 40.7% of the individuals had hypotension and decreased body weight, respectively [41]. Diet and foods containing extruded amaranth oil reduced total cholesterol, LDL-c and triglycerides by approximately 50%. Previously, very-low-density lipoprotein cholesterol (VLDL-c) concentrations were compared between hypercholesterolemia and control rabbits [42]. Among the hypercholesterolemia rabbits, those with remarkably low heart rate variability (HRV; total power (TP) 400 ms^2^) were assigned to the resistance-deficient group (Group 1), whereas those with a slightly higher HRV (TP > 400 ms^2^) were assigned to the low resistance group (Group 2). Regional and national level athletes with TP ranging from 3500 to 7000 ms^2^ were allocated to Group 3 [43]. Administration of amaranth oil at 18 mL per day for three weeks significantly lowered total cholesterol, triglycerides, LDL and VLDL-c in the subjects [37]. An LDL greater than 130 mg/dL, high-density lipoprotein (HDL) cholesterol lower than 35 mg/dL and total blood cholesterol greater than 200 mg/dL are indicators of high cholesterol, thereby marking a high risk of CVD development [44]. Both amaranth and quinoa seeds contain good quality of lutein, polyunsaturated fatty acids and tocopherols [45,46]; however, further research is required to confirm the effectiveness of these ingredients for CVD treatment in humans. In a previous prospective and double-blind study, postmenopausal women that consumed 25 g of quinoa flakes daily showed a decrease in total cholesterol and LDL-c, as well as an increase in GSH, which decreased their risk of CVD development [47].

### 3.3. Hepatitis

In a human study, a supplement containing uncooked red beet juice reduced non-HDL-c, LDL-c and total cholesterol [48]. In AML mice, treatment with betanin decreased LDL levels [49]. The consumption of a non-lipid diet increased serum TC, TC/HDL-c ratio, triglyceride (TAG) and atherogenic index, but decreased short-chain fatty acid (SCFA) production in rats [50]. However, the use of red beetroot (RBR) crisps inhibited the growth of TC and TAG, resulting in a higher probability of elevated total SCFA pool. The prescription of 3% RBR crisps also reduced the level of hepatic TC. Collectively, these findings suggest that the consumption of RBR crisps reduces metabolic changes in rats with dietary dyslipidemia [51]. However, another study on rats revealed that although RBR intake alleviated the concentration of SCFAs, it also caused the accumulation of long-chain fatty acids [52].

### 3.4. Antimicrobial and Antiviral Activities

**Figure 4:**
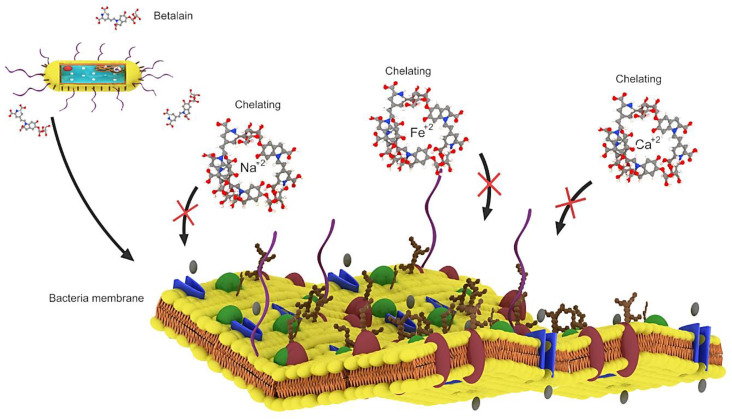
Betalain by chelating *Fe^+^*^2^******, *Ca^+^*^2^****** and *Mg^+^*^2^ ions which are among the basic needs of bacteria, betalain prevents them from entering the bacteria, resulting in the death of the bacteria.

Betalains exhibit antimicrobial and antimalarial effects, whereas betalain-rich *Amaranthus spinosus* shows prominent antimalarial activity in mice owing to its high levels of betanin and amaranthine, which can chelate the required inner cations (*Fe^+^*^2^*, Ca^+^*^2^ and *Mg^+^*^2^) and block the intracellular transport of choline in parasites [53]. Extracts of *Opuntia matudae*, which contain betalains, prevent the growth of *Escherichia coli* O157: H7 (Figure 4) [54]. Beetroot pomace induced a decrease in the growth of *Staphylococcus aureus*, *Salmonella typhimurium* and *Bacillus cereus* [55]. However, beetroot pomace was unable to prevent the growth of Gram-negative bacteria (*Pseudomonas aeruginosa*, *E. coli*, *Citrobacter freundii*, *Enterobacter cloacae*, *Salmonella typhimurium*, *Citrobacter youngae*), with *C. freundii* and *S. typhimurium* showing the highest susceptibility to beetroot pomace [56]. Betalain-rich extracts from red pitahaya exerted a broad-spectrum antimicrobial activity by preventing the growth of Gram-positive bacteria (*Escherichia faecalis*, *B. cereus*, *Listeria monocytogenes* and *S. aureus*) at 7.8 mg/mL, Gram-negative bacteria (*E. cloacae*, *Proteus vulgaris*, *Proteus mirabilis*, *P. aeruginosa*, *Salmonella typhi Ty2, Yersinia enterocolitica*, *Klebsiella pneumonia*, *Enterobacter aerogenes* and *E. coli*) at 15.6–62.5 mg/mL, yeasts (*Rhizoctonia solani* and *Candida albicans*) at 125–250 mg/mL and molds (*Aspergillus flavus*, *Cladosporium herbarium*, *Fusarium oxysporum* and *Botrytis cinerea*) at 500 mg/mL [57]. The antimicrobial activity of betalains is speculated to be caused by their negative effects on the function, structure and penetration of the microbial cell membrane, ultimately causing cell death [56]. Although betalains are known to exert broad-spectrum antimicrobial activity, only their microbial prevention mechanism was reported. The basic molecular and cellular mechanism underlying the antimicrobial effect of betalains will be highlighted in future studies [56].

### 3.5. Cognitive Impairment

Most of the people with cognitive impairment diseases such as dementia and Alzheimer’s disease suffer from cerebral circulatory disorders. Nitrate, which is metabolized and produced in beet nitric oxide (NO), has the ability to improve circulatory problems [58,59]. In a study of 75-year-old volunteers on a diet containing red beet juice, a significant increase in blood flow was observed by magnetic resonance imaging (MRI) of the brain in areas related to cognitive activity [60]. However, in other studies there were conflicting results related to the design and study groups selected. The betanine compound in red beet extract has been shown to help reduce the accumulation of inappropriate proteins in the brain (a process associated with Alzheimer’s disease). Ming and the authors showed that betanine is a promising compound for inhibiting adverse reactions in the brain that are involved in the progression of Alzheimer’s disease. Beta-amyloid is an adhesive fragment of a protein or peptide that accumulates in the brain and disrupts the connection between nerve synapses. This damage becomes more severe when amyloid beta binds to metals such as *Fe* and *Cu*. Metals lead to errors in the process of accumulation and accumulation of beta-amyloid protein, creating masses that cause inflammation and oxidation and ultimately the destruction of nerve cells. When betanine was added to the Cu-bound amyloid beta protein, oxidation was reduced by up to 90% and the folding abnormalities in the proteins stopped. Therefore, it seems that the main mechanism of betanine is the reduction of oxidation, which slows down the accumulation of beta-amyloid protein [61].

In Parkinson’s disease, 70–50% of dopaminergic neurons are significantly breaks down in the black liver [62]. As mentioned earlier, L-dopa is an intermediate compound in the red beet pigment production process. L-dopa is a major drug in the treatment of Parkinson’s disease that converts dopamine through the enzyme tyrosine hydroxylase [26,63]. In the Parkinson’s model of rats induced by tacrine, haloperidol and reserpine, administration of red beet (100, 200 and 300 mg/kg po) can protect against behavioral changes and its beneficial effects against Parkinson’s disease with antioxidant activity and possibly Show dopaminergic activity [64]. Of course, what has been mentioned requires more research.

### 3.6. Anticancer Activity

**Figure 5:**
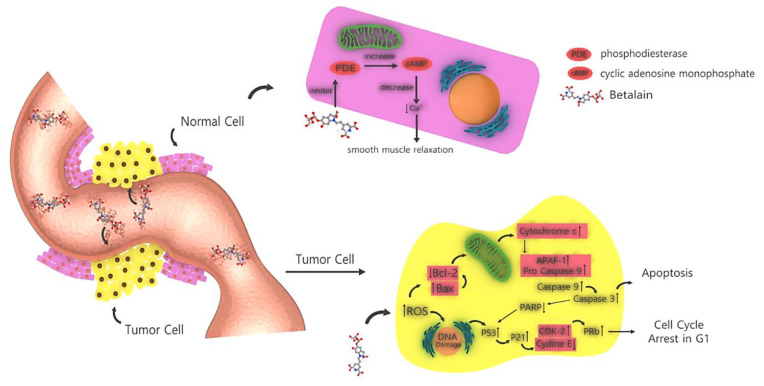
How betalain affects cancer cells and induces apoptosis in them vs. how betalain affects normal cells.

**Figure 6:**
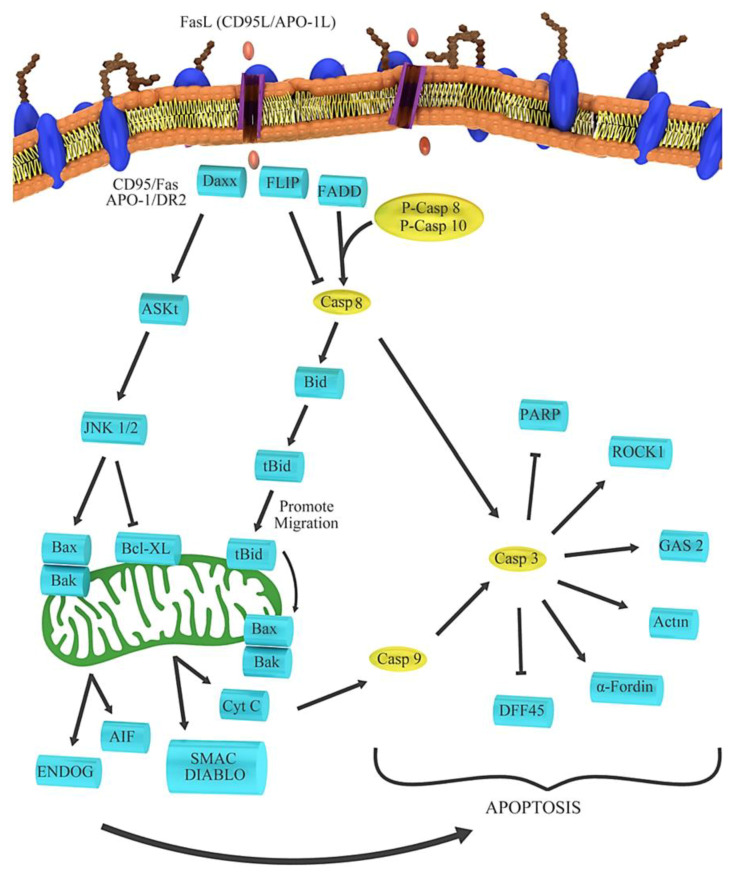
Processes involved in the *Apo-1* route.

There has been a growing interest in the anticancer properties of beets and the use of beet products or their ingredients as dietary supplements for cancer prevention [65]. Recent studies on betalain and their in vitro outcomes against cancer are presented in Table 2. Among the different atypical mechanisms underlying the chemopreventive attributes of beetroot at the cellular level, the anti-inflammatory, antioxidant, proapoptotic, antiproliferative and free radical-scavenging mechanisms have been investigated. Previous studies have shown marked increases in *BAX*, *caspase 9, caspase 3, cytochrome* and ROS as well as decreases in *BCL2* and *PARP*, causing DNA damage and ultimately leading to apoptosis. This process is shown in Figure 5 [66]. Beetroot is known for its high antioxidant activity, which is attributable to its pigments (i.e., betalains) [67]. The red components (betacyanins) of beetroot contain 75–95% betanin, which is considered to be its main pigment and the indicator of its phytochemical activity [68]. Although it is hypothesized that betanin is responsible for the beneficial effects of beet or beet fruit juice, cytotoxicity analysis revealed that the *p53* wild-type cancer cell lines (B16F10 and MCF-7) are highly sensitive to 40 μM of the betanin/isobetanin mixture (as indicated by inhibited proliferation and low cell resistance), whereas cancer cell lines (e.g., HT-29) expressing the less mutated *p53* (MDA-MB-231) are not sensitive to this mixture at the same concentration [69]. Because the effect of a betanin-rich extract was similar in both 2D and 3D culture conditions, the betanin/isobetanin concentrate was further found to inhibit the formation of a cluster, a cell structure that is resistant to apoptosis in cancer cell proliferation [70]. In the above process, MDA-MB-231 and B16F10 are metastatic cells cultured in the independent state of the anchor owing to the activation of the ERK signaling pathway. [71,72]. Therefore, the detection of an efficient molecule in anoikis-resistant cancer cells would be a promising objective in a future study. The inhibition of cell proliferation by mixture confirmed its anticancer properties and its effect on various cellular cycles. In MCF-7 cells, betanin/isobetanin extract reduced the number of G1-phase cells and increased the number of S-phase cells. It was also observed in MCF-7 cells treated with resveratrol or riproximin [73,74]. Betanin/isobetanin extract also contains cell cycle regulators, such as resveratrol and riproximin, which regulate the levels of cyclin A2 and cyclin B1 in MCF-7 cells. MDA-MB-231 cells cultured in 2D were arrested at the G1 phase after treatment with betanin/isobetanin extract. However, when these cells were cultured as aggregates, these molecules did not significantly affect the cell cycle progression. When assessing the cellular toxicity of red beet extract in MCF-7 cells, Cappadocia et al. found that the IC_50_ of the extract was 600 µmol (after 72 h of exposure) [66,75]. Reddy et al. [15] found inhibition of MCF-7 cell growth after treatment with betanin concentrate for 48 h (294 μM IC_50_). Overall, our findings were consistent with the results of the above studies. Betanin purified from raw beet extract significantly inhibited the growth of MCF-7, inducing cell death at very low concentrations (below 40 µM) [69]. Because the survival of MCF-7 cells was severely reduced by treatment with the betanin/isobetanin mixture, the nature of cell death was investigated. Using different methods, it was found that treatment with betanin causes apoptosis in 2DMCF-7 cells [69]. The expression of apoptotic proteins (*bad, TRAILR4, FAS* and *phosphorylated p53*) was dramatically increased and mitochondrial membrane potential was markedly altered [69]. Betanin decreases the number of small endothelial *CD31* vessels and increases the expression of *caspase 3*, indicating that its inhibitory effects on lung tumor is mediated through induction of apoptosis and inhibition of angiogenesis. Betanin also caused apoptosis by activating *caspases 3, 7, 9* and *PARP* in human lung cancer cell lines. Our data suggest that betanin significantly inhibits lung tumor growth in A/J mice and acts as a carcinogen in human lung cancer [76]. Previously, fluorescence-activated cell sorting analysis showed induction of apoptosis and increased activity of *caspases 3* and *8* [76]. RTqPCR assay showed that the combination of XVX + BC can increase the expression level of proapoptotic *BAX* and decrease the expression of anti-apoptotic anti-*BIRC5* (survivin) and pro-survival CTNNB1 (*β-catenin*) [77]. The most obvious effect of BC was an increase in *caspase 8* activity, which led to the induction of external apoptosis [77]. In the *APO-1* pathway, the apoptotic genes activate caspase 8. After that, the apoptotic pathway is activated by *caspase 3* and other genes involved in apoptosis, as shown in Figure 6. In another study, treatment with betanin/isobetanin resulted in a significant reduction in the proliferation and survival of cancer cells, changes in mitochondrial membrane potential (via both internal and external apoptosis pathways) and the formation of autophagous vesicles in MCF-7-treated cells [50]. In addition to a significant increase in the protein expression of *Bad*, *TRAILR4*, *FAS* and *p53*, the treatment led to autophagic cell death. The researchers concluded that betanin/isobetanin treatment may be useful for the treatment of cancer, especially in functional *p53* tumors. Although betanin-rich extract does not affect normal cell lines [69], betanin increased the proliferation of chronic human myeloid leukemia cell line (K562) in a dose- and time-dependent manner [50]. In addition, treatment with 40 mM betanin resulted in cells entering the phase below G0/G1 (28.4% of cells); the activation of apoptotic processes such as chromatin condensation, cellular contraction, membrane hemorrhage, DNA fragmentation and poly ribs (ADP) cleavage polymerization; reduction of membrane potential; regulation of *Bcl-2*; and release of cytochrome c into the cytosol [50]. Using confocal microscopy, betanin was observed to enter cells and induce apoptosis in K562 cells via intrinsic pathways [78].

## 4. Approaches to Enhance the Oral Bioavailability and Stability of Betalains

Bioavailability is defined as phytochemical percentage of a drug that enters the bloodstream [79]. The bioavailability of betalains has been reported in several animal and human studies. Netzel et al. [80] and Frank et al. [81] studied the pharmacokinetics of betalains in healthy humans after the ingestion of beet root juice. Postconsumption, betacyanins were immediately found in the urine; however, the amount of unmetabolized betalains excreted in urine was found to be significantly low. As the pigment content in urine accounted for 0.5–0.9 of the dose administered, the researchers concluded that renal clearance does not significantly aid in the systemic elimination of betalains [80]. It was hypothesized that other elimination pathways were involved, such as biliary excretion, enterohepatic circulation and metabolism, including metabolism by contributors such as intestinal bacteria [81]. Tesoriere et al. [82] simulated the gastric, oral and intestinal digestion of betalains in vitro by comparing various types of food consisting of pigment content. Their findings indicated that the food matrix prevented the degradation of betanin/isobetanin in the gastric environment. Furthermore, loss of betacyanins during digestion was observed in the small intestine, with differences observed for foods containing pigments and those containing purified betalains. Results showed that betalamic acid accumulation was observed after the degradation of purified betalains, however, this was not occur during the digestion of food containing betalains [82]. Therefore, the researchers concluded that the bioavailability of dietary betalains heavily depends on the chemical stability of the digestive tract; however, other factors such as the type of food matrix can alter the bioaccessibility of digestive enzymes [82]. Intestinal bacteria also participates actively in the metabolism of betalains and interfere with their absorption and bioavailability [83]. Tesoriere et al. [84] examined the permeability of red beet indicaxanthin and betanin in Caco-2 monolayer cells. Indicaxanthin was found to have a higher permeability coefficient than betanin. Further, the key step in the absorption of betalains was attributed to multidrug resistance-associated protein-2 (*MRP-2*), which controls the efflux of phytochemicals via a dose-dependent activity [84].

**Figure 7:**
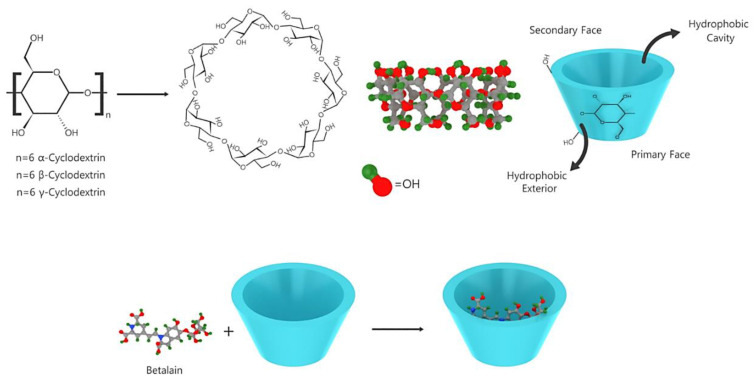
Betalain encapsulated by cyclodextrin.

β-cyclodextrin and glucose oxidase contribute to betalain stabilization via the adsorption of free water and the removal of dissolved oxygen, respectively [85,86]. Interestingly, phenolic antioxidants and tocopherol did not exhibit any stabilizing effect on betalain [87]. Because of the conjugated dienes in the 1,7-diazaheptamethine structure, betalains absorb UV and visible lights [88]. Previously, structural implication on the fluorescence of betaxanthins has been reported [89]. In addition to the use of antioxidants, the metal chelating agent EDTA and inclusion complexes containing maltodextrin and β-cyclodextrin, encapsulation is an efficient method to stabilize and ease the administration of betalain. As shown in Figure 7, the effect of encapsulation on the stabilization and improvement of the bioavailability of polyphenols has been previously investigated [90,91].

Collectively, these data confirm the high availability of betalains in the human body, with betaxanthin showing greater bioavailability than betacyanin. However, further research is necessary to elucidate the specific content of betalain metabolites in plasma, urine and bile [92]. Owing to its high bioavailability and health-protective effect, betaxanthin has been employed as a food supplement to enhance the quality of processed food products [93]. Studies regarding the stability and bioaccessibility of betalains under simulated digestive conditions propose that digestive stability manage the bioaccessibility of betaxanthins, whereas additional factors relevant to the food matrix and food processing affect betacyanin bioaccessibility. Previously, the radical-scavenging activity and stability of betalains under simulated human gastrointestinal tract conditions have been examined [94]. When the pH value was less than three and the concentration of bile salts was increased to 4%, betalains were relatively stable and their radical-scavenging activities decreased from 75% to 38%. Similarly, the antiradical activities and stabilities of betanin under different pH, temperature, and light conditions have been previously examined [90].

## 5. Conclusions and Future Trends

Numerous studies have revealed the health benefits of betalains arising from their high antioxidant capacity (Table 2 and Table 3). Although betalains were previously restricted to plants belonging to the order Caryophyllales and some fungal species, the present study revealed the first betalain-producing bacteria as well as the main steps involved in the pigment formation. Moreover, our findings indicate that the biosynthesis of betalain can be extended to prokaryotes. Betalains are formed through decisive steps in the biosynthesis of beta-beta; these include the condensation of the beta-chromatin chromophore, betalamic acid, with cyclo-dopa and amino acids or amino acids alone or those involved in the formation of the corresponding aldimine from the red-purple beta and yellow betaxanthins. Because of their use as food colorants, antiseptics and radioactive radicals to protect against stress-related disorders, betalain enzymes have attracted the attention of researchers. However, future studies on pure beta-lysine are needed to elucidate more thoroughly its precise biologic functions.

**Table 2:** Outcome and purpose of recent in vitro studies on cancer therapy of betalain.

Source of betalain	Type of study	Applications	Outcomes	Ref.
*Celosia argentea* var. *plumosa*	In vitro	Production of betalains	Production of dihydroxylated betalains in the cells during eight days of culture	[95]
*Lepismium lorentzianum*, *Lepismium lumbricoides*, *Rhipsalis floccosa* and *Pfeiffera ianthothele*	In vitro	Antimutagenic	Significant antimutagenic effect for *L. lumbricoides* and weak effect for *P. ianthothele* and *R. floccosa*	[96]
*Opuntia* spp.	In vitro (various cell lines)	Anticancer	Among the cancer lines tested, the viability of prostate and colon cells was the most affected	[97]
*Beta vulgaris* (beetroot)	In vitro Lung cancer (A549), human prostate (PC-3) and breast (MCF-7 and MDA-MB-231) cancer cell lines	Anticancer	Beetroot ingestion can be used to prevent cancer Betanin may contribute to the cytotoxicity and chemo preventive activities of beetroot extract when used alone or in combination with doxorubicin to mitigate the toxic side effects of the latter	[76,98]
	Ex vivo (Rat skin and lung tissues)			
*Opuntia* spp.	In vitro Human colon cancer cell line (HT29)	Antiproliferative	An unexpected increase in intracellular ROS accumulation in HT29 cells suggested that cancer cell death may be induced by the pro-oxidant effect	[99]
*O. ficus-indica*	In vitro Chronic myeloid leukemia cell line (K562)	Anticancer	Betanin induced apoptosis in K562 cells through the intrinsic pathway and this was mediated by the release of cytochrome c from the mitochondria into the cytosol as well as by PARP cleavage	[78]

## Figures and Tables

**Table 1:** Betalain-producing plant species.

Family	Species	Common Name or Representative	Chemical Structures	Betalains	References
Achatocarpaceae Aizoaceae	*–*	–	–	–	[17]
Amaranthaceae	*Amaranthus spinosus*		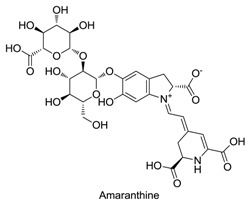	Amaranthine, isoamaranthine	[18]
	*Gomphrena globosa*	Spiny amaranth	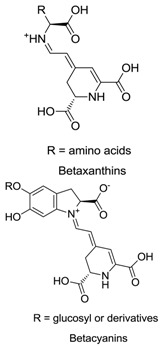	Betaxanthins and several betacyanins	[19]
	*Celosia argentea* (*var. plumose* and *var. cristata*)	Feathered amaranth and common cockscomb	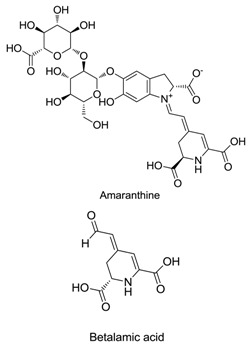	Amaranthine, betalamic acid, dopamine-derived betacyanins	[20]
Cactaceae	*Hylocereus polyrhizus*	Red-purple pitaya	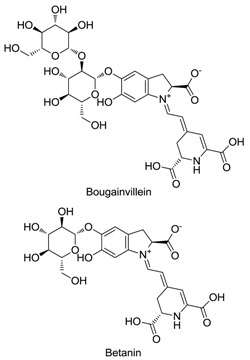	Betacyanins (10 kinds), bougainvillein-r-I, betanin, isobetanin, phyllocactin, isophyllocactin, hylocerenin	[21,22]
Aizoaceae	*Lampranthus productus*	Ice plant	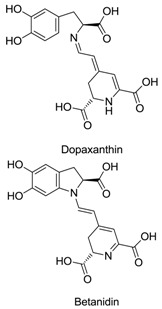	Dopaxanthin, betanidin	[23]
Nyctaginaceae	*Boerhavia erecta*	Erect spiderling	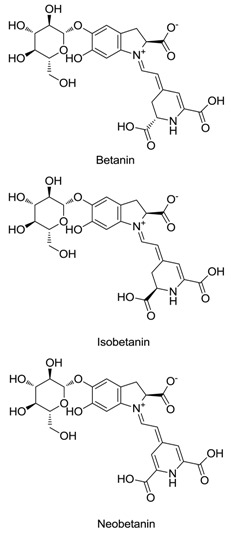	Betanin, isobetanin, neobetanin	[24]
Portulacaceae	*Portulaca grandiflora*	Moss rose	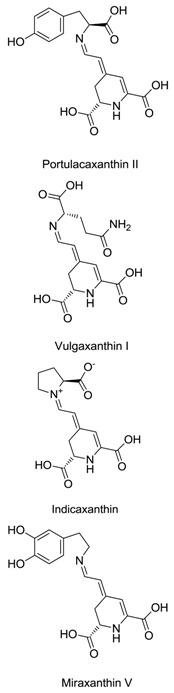	Dopaxanthin, portulacaxanthin II, vulgaxanthin I, miraxanthin V, indicaxanthin	[25,26]

**Table 3:** Pharmacological benefits of betalains.

Therapeutic Application	Source of Betalain		Outcomes	References
	Species	Active Components/Parts		
Antidiabetic	Beet	–	Experiments have shown a 40% reduction in glycemia, without weight loss and liver dysfunction. The action of hypoglycemia mechanism for the extract is experimentally attributed to saponins that inhibit glycogenolysis and gluconeogenesis.	[100]
		–	Reduce serum glucose, lipid profile, ALT, AST, TNF-α, IL-1β, IL-6, MDA and increase in hepatic TAO and GST in rats.	[101]
	Red beetroot	Betavulgaroside I, II, III and IV	Reduce blood glucose in rats.	[102]
		Betalains	Reduce blood glucose levels in mice.	[103]
		Betanin	Prevent induction of diabetes by alloxan in mice; reduce cardiogenic fibrosis in rats.	[104,105,106]
		Apigenin	Increase insulin level in mice.	[107]
		Luteolin	Increase insulin level in mice.	[108]
		Quercetin	Decrease blood glucose level in rats.	[109]
		Kaempferitrin	Increase antioxidant and hypoglycemic effects in rats.	[110]
		Epicatechin	Revive insulin-producing cells in rats.	[111]
		–	Inhibit absorption and digestion of glucose in intestine in mice.	[103]
		Aqueous extract	Increase glucose disposal in skeletal myocytes and glucose absorption through GLUT4 transporters in mice.	[112]
	Chard	Aqueous chard extract	Increase number and volume of secretion of insulin-producing cells in humans.	[28]
Cardiovascular disease	Red beetroot	Nitrate in red beet –	Reduce the blood pressure and LDL cholesterol in humans.	[113]
		–	Reduce serum total cholesterol and triacylglycerol levels in rats.	[51]
		Pulp	Reduce cholesterol and triglycerides in rats.	[114]
		–	improve in early vascular dysfunction and Reduce LDL cholesterol levels, increase HDL cholesterol levels; reduce oxidative stress; invert injury to brachial endothelial artery, improve function of the muscles and increase strength; reduce systolic blood pressure (4–5 mmHg); increase antithrombotic, antiadhesive effects; reduce blood pressure and improve brachial artery blood flow in humans.	[115,116,117]
		Ethanol extract of stalks and leaves	Reduce oxidative stress, blood glucose and cholesterol in liver in mice.	[118]
		Fiber content in the red beet	Reduce cholesterol and the number of tumors of colon cancer in rats.	[119]
		Betanin	Temporarily increase heart rate and blood pressure in rats; increase SIRT1 and reduce LOX1 and hs-CRP in humans.	[50,120]
Anti-hepatitis	*Boerhavia diffusa L.*	Spongy roots decoction	–	[121]
	*B. diffusa*	Root extract	According to studies, *B. diffusa* showed the potential to cure infectious hepatitis by antiviral mechanism. In the study, *B. diffusa* root extract (5 mg/mL) showed antiviral potency by inhibiting surface antigen as well as inhibiting HBV (hepatitis B virus).	[122]
Antibacteria	*B. diffusa*	Methanolic extract	The ethanolic extract of whole plant of *B. diffusa* has antimicrobial activity against bacterial strains *Bacillus subtilis* UC564, *Staphylococcus aureus* 15 ML296, *Staphylococcus aureus* ML329 and *Salmonella typhi* DI at 2000 µg/mL.	[121]
	*Opuntia matudae*	Extract of whole plant	*Opuntia matudae* extract has the potential to inhibit the growth four strains of *E. coli* O157:H7 and could provide a natural means of controlling pathogenic contamination.	[54]
	*Hylocereus polyrhizus*	Subfractionation extract	Flesh and peels extract have wide range of antimicrobials spectrum to prevent the growth of all pathogenic bacteria and/or human food spoilage, molds and yeasts.	[57]
Cognitive improvement	Red beetroot	–	Increase blood supply the brain in areas related to cognitive activity in humans.	[60]
Alzheimer’s disease		Betanin	Reduce accumulation of β-amyloid protein in humans.	[123]
Parkinson’s		Methanolic extract	Increase antioxidant activity and possible dopaminergic activity in rats.	[65]
Anticancer				Table 2
